# [3-(5-Hy­droxy-5*H*-dibenzo[*a*,*d*]cyclo­hepten-5-yl)prop­yl]dimethyl­ammonium 3-carboxyprop-2-enoate

**DOI:** 10.1107/S1600536811036257

**Published:** 2011-09-14

**Authors:** Jerry P. Jasinski, James A. Golen, M. S. Siddegowda, H. S. Yathirajan, B. Narayana

**Affiliations:** a Department of Chemistry, Keene State College, 229 Main Street, Keene, NH 03435-2001, USA; b Department of Studies in Chemistry, University of Mysore, Manasagangotri, Mysore 570 006, India; c Department of Studies in Chemistry, Mangalore University, Mangalagangotri, 574 199, India

## Abstract

In the cation of the title salt, C_20_H_24_NO^+^·C_4_H_3_O_4_^−^, the N atom in the dimethyl­ammonium group is protonated. The dihedral angle between the mean planes of the two six-membered rings fused to the cyclo­hepten-5-yl ring is 54.4 (1)°. An intra­molecular O—H⋯O hydrogen bond occurs in the anion. The crystal packing is stabilized by inter­molecular O—H⋯O and N—H⋯(O,O) hydrogen bonds and weak C—H⋯O inter­actions, forming a two-dimensional network.

## Related literature

The title compound is used in the preparation of cyclo­benzaprine (systematic name: 3-(5*H*-dibenzo[*a*,*d*]cyclo­hepten-5-yl­idene)-*N*,*N*-dimethyl-1-propanamine), a muscle relaxant used to relieve skeletal muscle spasms and associated pain in acute musculoskeletal conditions. For its structural relationships to first-generation tricyclic anti­depressants, see: Com­miss­iong *et al.* (1981 ▶); Katz & Dube (1988 ▶); Cimolai (2009 ▶). For related structures, see: Bindya *et al.* (2007 ▶); Jasinski, Pek *et al.* (2010 ▶); Jasinski, Butcher *et al.* (2010 ▶); Fun *et al.* (2011 ▶); Siddegowda *et al.* (2011 ▶). For standard bond lengths, see: Allen *et al.* (1987 ▶).
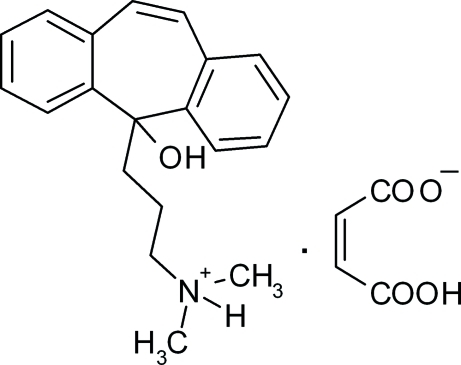


## Experimental

### 

#### Crystal data

C_20_H_24_NO^+^·C_4_H_3_O_4_^−^
*M* *_r_* = 409.47
Monoclinic, 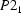

*a* = 9.2115 (2) Å
*b* = 11.5840 (2) Å
*c* = 10.4640 (2) Å
β = 101.591 (2)°
*V* = 1093.80 (4) Å^3^
*Z* = 2
Mo *K*α radiation
μ = 0.09 mm^−1^
*T* = 173 K
0.40 × 0.22 × 0.20 mm

#### Data collection

Oxford Diffraction Xcalibur Eos Gemini diffractometer
Absorption correction: multi-scan (*CrysAlis RED*; Oxford Diffraction, 2010 ▶) *T*_min_ = 0.966, *T*_max_ = 0.983
9674 measured reflections
2834 independent reflections
2683 reflections with *I* > 2σ(*I*)
*R*_int_ = 0.016

#### Refinement

*R*[*F* ^2^ > 2σ(*F* ^2^)] = 0.033
*wR*(*F* ^2^) = 0.093
*S* = 1.04
2834 reflections
282 parameters
4 restraints
H atoms treated by a mixture of independent and constrained refinement
Δρ_max_ = 0.33 e Å^−3^
Δρ_min_ = −0.26 e Å^−3^

### 

Data collection: *CrysAlis PRO* (Oxford Diffraction, 2010 ▶); cell refinement: *CrysAlis PRO*; data reduction: *CrysAlis RED* (Oxford Diffraction, 2010 ▶); program(s) used to solve structure: *SHELXS97* (Sheldrick, 2008 ▶); program(s) used to refine structure: *SHELXL97* (Sheldrick, 2008 ▶); molecular graphics: *SHELXTL* (Sheldrick, 2008 ▶); software used to prepare material for publication: *SHELXTL*.

## Supplementary Material

Crystal structure: contains datablock(s) global, I. DOI: 10.1107/S1600536811036257/bt5634sup1.cif

Structure factors: contains datablock(s) I. DOI: 10.1107/S1600536811036257/bt5634Isup2.hkl

Supplementary material file. DOI: 10.1107/S1600536811036257/bt5634Isup3.cml

Additional supplementary materials:  crystallographic information; 3D view; checkCIF report

## Figures and Tables

**Table 1:** Hydrogen-bond geometry (Å, °)

*D*—H⋯*A*	*D*—H	H⋯*A*	*D*⋯*A*	*D*—H⋯*A*
O1—H1*O*⋯O3^i^	0.83 (2)	1.95 (2)	2.770 (2)	173 (2)
O2—H2*O*⋯O4	0.89 (2)	1.56 (2)	2.442 (2)	171 (4)
N1—H1*N*⋯O5	0.88 (2)	1.80 (2)	2.6797 (19)	172 (2)
N1—H1*N*⋯O4	0.88 (2)	2.69 (2)	3.340 (2)	131 (2)
C16—H16*B*⋯O3^i^	0.99	2.63	3.267 (3)	122
C19—H19*A*⋯O3^ii^	0.98	2.55	3.452 (3)	154
C20—H20*A*⋯O3^ii^	0.98	2.94	3.781 (4)	144
C9—H9*A*⋯O4^iii^	0.95	2.82	3.675 (2)	151
C12—H12*A*⋯O4^iv^	0.95	2.62	3.460 (3)	148
C17—H17*A*⋯O5^v^	0.99	2.92	3.865 (2)	159
C20—H20*B*⋯O5^v^	0.98	2.39	3.296 (3)	154

Symmetry codes: (i) 
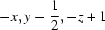
; (ii) 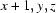; (iii) 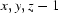; (iv) 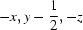; (v) 
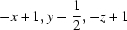
.

**Fig. 1.**
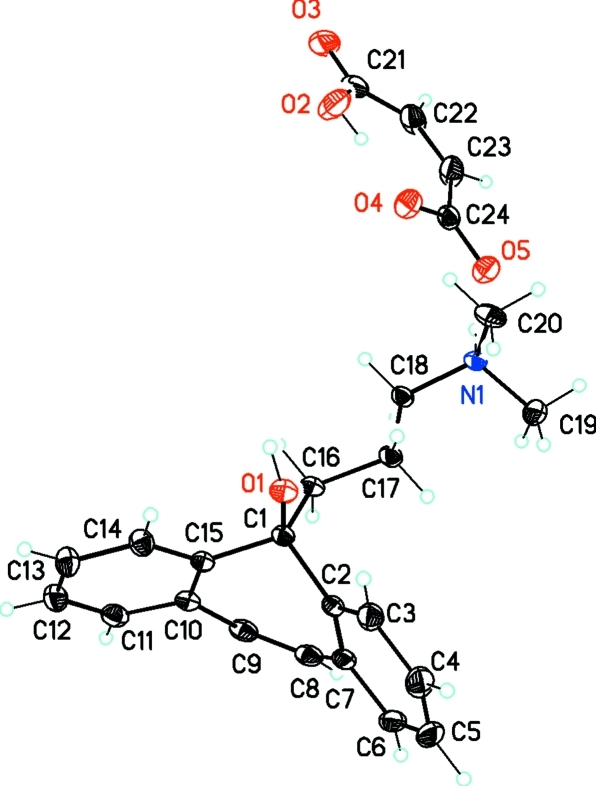
Molecular structure of the title compound, showing the atom-labeling scheme and 30% probability displacement ellipsoids.

**Fig. 2.**
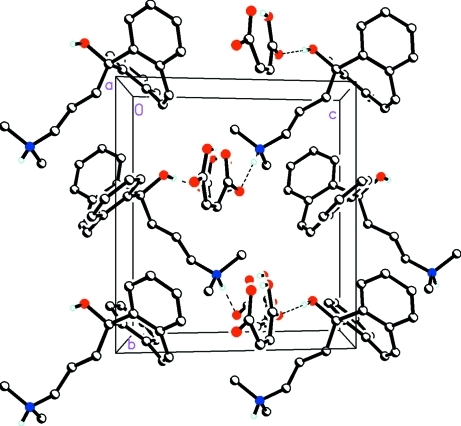
Packing diagram of the title compound, viewed down the a axis. Dashed lined indicate N—H···O and O—H···O intermolecular hydrogen bonds forming a 2-D network.

## supplementary crystallographic information

### Comment

The title compound is used for the preparation of cyclobenzaprine. Cyclobenzaprine (Systematic iupac name: 3-(5H-dibenzo[a,d]cyclohepten-5- ylidene)-N,N-dimethyl-1-propanamine) is a muscle relaxant used to relieve skeletal muscle spasms and associated pain in acute musculoskeletal conditions.  Cyclobenzaprine has been considered structurally related to the first-generation tricyclic antidepressants (Commissiong *et al.*, 1981; Katz & Dube, 1988; Cimolai, 2009). The crystal structures of amitriptylinium picrate (Bindya *et al.*, 2007), 4-(4-chlorophenyl)-4-hydroxypiperidinium maleate maleic acid solvate (Jasinski, Pek *et al.*, 2010), trimipraminium maleate (Jasinski, Butcher  *et al.*, 2010), cyclobenzaprinium salicylate (Fun *et al.*, 2011) and cyclobenzaprinium chloride (Siddegowda *et al.*, 2011) have been reported. In view of the importance of 3-(5-hydroxy-5H-dibenzo[a,d] cyclohepten-5-yl)-propyl]-dimethylammonium maleate, this paper reports the crystal structure of the title salt, (I), C_20_H_24_NO^+^.C_4_H_3_O_4_^-^.

In the cation of the title salt, C_20_H_24_NO^+^.C_4_H_3_O_4_^-^, the N atom in the dimethylammonium group is protonated (Fig 1).  The dihedral angle between the mean planes of the two benzene rings fused to the seven-membered cyclohepten-5-yl ring is 54.4 (1)°.  Crystal packing is stabilized by O—H···O, N—H···O intermolecular hydrogen bonds, N—H···O intramolecular bonds and weak C—H···O intermolecular interactions (Table 1) forming a 2-D network (Fig. 2).

### Experimental

3-(5-Hydroxy-5H-dibenzo[a,d]cyclohepten-5-yl)-propyl]-dimethylamine (2.0 g, 0.0068 mol) and maleic acid (0.788 g, 0.0068 mol) were dissolved in 10 ml of ethyl acetate taken in a 50 ml round bottomed flask. The reaction mixture was heated to 323-333 K with constant stirring for 30 min.  The product formed was filtered, dried and recrystallized from methanol (m.p.: 419-421 K).

### Refinement

H1O and H1N were located by a Fourier map and refined isotropically.  All of the remaining H atoms were placed in their calculated positions and then refined using the riding model with C–H lengths of 0.95 Å (CH), 0.99 Å (CH_2_) or 0.98 Å (CH_3_).  The isotropic displacement parameters for these atoms were set to 1.19–1.21 (CH), 1.18–1.19 (CH_2_) or 1.50–1.51 (CH_3_) times *U*_eq_ of the parent atom. In the absence of anomalous scatterers,  2834 Friedel pairs were merged.

### Crystal data

**Table:**

C_20_H_24_NO^+^·C_4_H_3_O_4_^−^	*F*(000) = 436
*M**_r_* = 409.47	*D*_x_ = 1.243 Mg m^−^^3^
Monoclinic, *P*2_1_	Mo *K*α radiation, λ = 0.71073 Å
Hall symbol: P 2yb	Cell parameters from 5270 reflections
*a* = 9.2115 (2) Å	θ = 3.2–32.2°
*b* = 11.5840 (2) Å	µ = 0.09 mm^−^^1^
*c* = 10.4640 (2) Å	*T* = 173 K
β = 101.591 (2)°	Block, colorless
*V* = 1093.80 (4) Å^3^	0.40 × 0.22 × 0.20 mm
*Z* = 2	

### Data collection

**Table:**

Oxford Diffraction Xcalibur Eos Gemini diffractometer	2834 independent reflections
Radiation source: Enhance (Mo) X-ray Source	2683 reflections with *I* > 2σ(*I*)
graphite	*R*_int_ = 0.016
ω scans	θ_max_ = 28.3°, θ_min_ = 3.2°
Absorption correction: multi-scan (*CrysAlis RED*; Oxford Diffraction, 2010)	*h* = −12→9
*T*_min_ = 0.966, *T*_max_ = 0.983	*k* = −15→15
9674 measured reflections	*l* = −13→13

### Refinement

**Table:**

Refinement on *F*^2^	Primary atom site location: structure-invariant direct methods
Least-squares matrix: full	Secondary atom site location: difference Fourier map
*R*[*F*^2^ > 2σ(*F*^2^)] = 0.033	Hydrogen site location: inferred from neighbouring sites
*wR*(*F*^2^) = 0.093	H atoms treated by a mixture of independent and constrained refinement
*S* = 1.04	*w* = 1/[σ^2^(*F*_o_^2^) + (0.0573*P*)^2^ + 0.1521*P*] where *P* = (*F*_o_^2^ + 2*F*_c_^2^)/3
2834 reflections	(Δ/σ)_max_ = 0.002
282 parameters	Δρ_max_ = 0.33 e Å^−^^3^
4 restraints	Δρ_min_ = −0.26 e Å^−^^3^

### Special details

**Table:**

Geometry. All esds (except the esd in the dihedral angle between two l.s. planes) are estimated using the full covariance matrix. The cell esds are taken into account individually in the estimation of esds in distances, angles and torsion angles; correlations between esds in cell parameters are only used when they are defined by crystal symmetry. An approximate (isotropic) treatment of cell esds is used for estimating esds involving l.s. planes.
Refinement. Refinement of *F*^2^ against ALL reflections. The weighted *R*-factor *wR* and goodness of fit *S* are based on *F*^2^, conventional *R*-factors *R* are based on *F*, with *F* set to zero for negative *F*^2^. The threshold expression of *F*^2^ > σ(*F*^2^) is used only for calculating *R*-factors(gt) *etc*. and is not relevant to the choice of reflections for refinement. *R*-factors based on *F*^2^ are statistically about twice as large as those based on *F*, and *R*- factors based on ALL data will be even larger.

### Fractional atomic coordinates and isotropic or equivalent isotropic displacement parameters (Å^2^)

**Table:**

	*x*	*y*	*z*	*U*_iso_*/*U*_eq_	
O1	0.41330 (14)	0.34062 (11)	0.16548 (12)	0.0302 (3)	
H1O	0.348 (2)	0.353 (2)	0.208 (2)	0.036*	
O2	−0.0516 (2)	0.7602 (3)	0.6263 (2)	0.0758 (7)	
H2O	0.037 (3)	0.759 (4)	0.606 (3)	0.091*	
O3	−0.2140 (2)	0.8844 (3)	0.6744 (2)	0.0924 (10)	
O4	0.18355 (17)	0.77043 (15)	0.55298 (16)	0.0460 (4)	
O5	0.32094 (17)	0.90841 (13)	0.49397 (16)	0.0450 (3)	
N1	0.46816 (15)	0.73787 (12)	0.40245 (12)	0.0244 (3)	
H1N	0.412 (2)	0.7907 (18)	0.430 (2)	0.029*	
C1	0.39082 (17)	0.42003 (14)	0.06008 (14)	0.0240 (3)	
C2	0.53853 (18)	0.42751 (15)	0.01408 (16)	0.0277 (3)	
C3	0.6595 (2)	0.36330 (18)	0.0782 (2)	0.0372 (4)	
H3A	0.6485	0.3144	0.1485	0.045*	
C4	0.7959 (2)	0.3695 (2)	0.0410 (3)	0.0502 (6)	
H4A	0.8766	0.3244	0.0852	0.060*	
C5	0.8143 (2)	0.4409 (2)	−0.0598 (3)	0.0527 (6)	
H5A	0.9076	0.4455	−0.0850	0.063*	
C6	0.6963 (3)	0.5059 (2)	−0.1237 (2)	0.0455 (5)	
H6A	0.7101	0.5559	−0.1922	0.055*	
C7	0.5557 (2)	0.49996 (16)	−0.09015 (18)	0.0328 (4)	
C8	0.4376 (2)	0.56887 (17)	−0.16865 (17)	0.0366 (4)	
H8A	0.4668	0.6404	−0.2000	0.044*	
C9	0.2936 (2)	0.54318 (16)	−0.20162 (17)	0.0348 (4)	
H9A	0.2312	0.5988	−0.2522	0.042*	
C10	0.22200 (19)	0.43761 (15)	−0.16756 (16)	0.0284 (3)	
C11	0.1030 (2)	0.39442 (19)	−0.26041 (17)	0.0369 (4)	
H11A	0.0649	0.4393	−0.3357	0.044*	
C12	0.0399 (2)	0.2891 (2)	−0.2456 (2)	0.0423 (5)	
H12A	−0.0394	0.2610	−0.3106	0.051*	
C13	0.0926 (2)	0.22432 (19)	−0.1354 (2)	0.0419 (4)	
H13A	0.0516	0.1505	−0.1253	0.050*	
C14	0.2057 (2)	0.26732 (17)	−0.03967 (18)	0.0336 (4)	
H14A	0.2395	0.2229	0.0368	0.040*	
C15	0.27125 (18)	0.37390 (14)	−0.05244 (15)	0.0253 (3)	
C16	0.34051 (18)	0.53637 (14)	0.10869 (15)	0.0257 (3)	
H16A	0.3252	0.5924	0.0357	0.031*	
H16B	0.2442	0.5252	0.1352	0.031*	
C17	0.45116 (19)	0.58700 (16)	0.22342 (16)	0.0293 (3)	
H17A	0.4873	0.5258	0.2880	0.035*	
H17B	0.5373	0.6196	0.1925	0.035*	
C18	0.37420 (18)	0.68131 (15)	0.28617 (15)	0.0269 (3)	
H18A	0.3389	0.7413	0.2198	0.032*	
H18B	0.2860	0.6476	0.3127	0.032*	
C19	0.5987 (2)	0.79753 (19)	0.3701 (2)	0.0382 (4)	
H19A	0.6437	0.8475	0.4429	0.057*	
H19B	0.5672	0.8444	0.2914	0.057*	
H19C	0.6713	0.7401	0.3546	0.057*	
C20	0.5116 (3)	0.65885 (18)	0.51531 (18)	0.0389 (4)	
H20A	0.5644	0.7025	0.5907	0.058*	
H20B	0.5763	0.5981	0.4931	0.058*	
H20C	0.4226	0.6238	0.5367	0.058*	
C21	−0.0942 (3)	0.8652 (3)	0.6456 (2)	0.0626 (8)	
C22	0.0010 (3)	0.9628 (3)	0.6312 (3)	0.0649 (8)	
H22A	−0.0342	1.0349	0.6559	0.078*	
C23	0.1278 (2)	0.9692 (2)	0.5899 (3)	0.0547 (7)	
H23A	0.1684	1.0446	0.5898	0.066*	
C24	0.2169 (2)	0.87546 (18)	0.54356 (19)	0.0359 (4)	

### Atomic displacement parameters (Å^2^)

**Table:**

	*U*^11^	*U*^22^	*U*^33^	*U*^12^	*U*^13^	*U*^23^
O1	0.0362 (6)	0.0289 (6)	0.0263 (6)	0.0060 (5)	0.0077 (5)	0.0048 (5)
O2	0.0552 (11)	0.1038 (19)	0.0738 (13)	−0.0219 (13)	0.0254 (10)	0.0128 (14)
O3	0.0507 (10)	0.174 (3)	0.0613 (11)	−0.0215 (15)	0.0322 (9)	−0.0421 (16)
O4	0.0427 (8)	0.0403 (8)	0.0564 (9)	−0.0014 (6)	0.0132 (7)	0.0013 (7)
O5	0.0435 (7)	0.0345 (7)	0.0622 (9)	−0.0019 (6)	0.0230 (7)	−0.0127 (7)
N1	0.0313 (7)	0.0222 (6)	0.0213 (6)	0.0016 (5)	0.0087 (5)	−0.0019 (5)
C1	0.0290 (7)	0.0213 (7)	0.0223 (7)	0.0007 (6)	0.0066 (5)	−0.0008 (6)
C2	0.0313 (8)	0.0245 (7)	0.0292 (7)	−0.0030 (6)	0.0104 (6)	−0.0104 (6)
C3	0.0328 (9)	0.0362 (9)	0.0422 (10)	0.0017 (7)	0.0069 (7)	−0.0097 (8)
C4	0.0315 (9)	0.0550 (13)	0.0647 (14)	0.0034 (9)	0.0110 (9)	−0.0216 (11)
C5	0.0373 (10)	0.0592 (14)	0.0688 (15)	−0.0114 (10)	0.0283 (10)	−0.0299 (12)
C6	0.0548 (12)	0.0439 (11)	0.0461 (11)	−0.0196 (10)	0.0302 (10)	−0.0193 (9)
C7	0.0414 (9)	0.0294 (8)	0.0313 (8)	−0.0092 (7)	0.0165 (7)	−0.0126 (7)
C8	0.0594 (11)	0.0265 (8)	0.0287 (8)	−0.0071 (8)	0.0204 (8)	−0.0012 (6)
C9	0.0536 (11)	0.0287 (8)	0.0235 (7)	0.0044 (8)	0.0111 (7)	0.0042 (6)
C10	0.0332 (8)	0.0297 (8)	0.0239 (7)	0.0045 (7)	0.0091 (6)	−0.0015 (6)
C11	0.0353 (9)	0.0484 (11)	0.0266 (8)	0.0060 (8)	0.0051 (6)	−0.0022 (8)
C12	0.0317 (9)	0.0552 (13)	0.0382 (10)	−0.0044 (9)	0.0026 (7)	−0.0119 (9)
C13	0.0414 (10)	0.0364 (10)	0.0476 (11)	−0.0099 (8)	0.0083 (8)	−0.0076 (9)
C14	0.0370 (9)	0.0281 (8)	0.0349 (9)	−0.0015 (7)	0.0051 (7)	−0.0008 (7)
C15	0.0273 (7)	0.0253 (7)	0.0243 (7)	0.0024 (6)	0.0080 (5)	−0.0029 (6)
C16	0.0316 (8)	0.0253 (7)	0.0209 (7)	0.0045 (6)	0.0070 (6)	−0.0025 (6)
C17	0.0286 (7)	0.0315 (8)	0.0284 (8)	0.0035 (6)	0.0073 (6)	−0.0091 (6)
C18	0.0283 (7)	0.0278 (8)	0.0246 (7)	0.0028 (6)	0.0053 (6)	−0.0061 (6)
C19	0.0383 (9)	0.0371 (10)	0.0408 (10)	−0.0093 (8)	0.0113 (8)	−0.0027 (8)
C20	0.0598 (11)	0.0346 (9)	0.0230 (8)	0.0057 (9)	0.0101 (7)	0.0043 (7)
C21	0.0449 (12)	0.111 (3)	0.0342 (10)	−0.0096 (15)	0.0146 (9)	−0.0235 (13)
C22	0.0394 (11)	0.092 (2)	0.0651 (15)	0.0005 (12)	0.0148 (10)	−0.0473 (16)
C23	0.0382 (10)	0.0539 (14)	0.0739 (16)	−0.0045 (10)	0.0158 (10)	−0.0352 (12)
C24	0.0314 (8)	0.0394 (10)	0.0366 (9)	0.0002 (8)	0.0059 (7)	−0.0117 (8)

### Geometric parameters (Å, °)

**Table:**

O1—C1	1.4191 (19)	C10—C11	1.403 (2)
O1—H1O	0.826 (16)	C10—C15	1.408 (2)
O2—C21	1.306 (5)	C11—C12	1.374 (3)
O2—H2O	0.885 (19)	C11—H11A	0.9500
O3—C21	1.221 (3)	C12—C13	1.379 (3)
O4—C24	1.264 (3)	C12—H12A	0.9500
O5—C24	1.238 (2)	C13—C14	1.385 (3)
N1—C19	1.484 (2)	C13—H13A	0.9500
N1—C20	1.484 (2)	C14—C15	1.392 (2)
N1—C18	1.495 (2)	C14—H14A	0.9500
N1—H1N	0.884 (16)	C16—C17	1.527 (2)
C1—C2	1.534 (2)	C16—H16A	0.9900
C1—C15	1.537 (2)	C16—H16B	0.9900
C1—C16	1.544 (2)	C17—C18	1.521 (2)
C2—C3	1.394 (3)	C17—H17A	0.9900
C2—C7	1.410 (3)	C17—H17B	0.9900
C3—C4	1.390 (3)	C18—H18A	0.9900
C3—H3A	0.9500	C18—H18B	0.9900
C4—C5	1.377 (4)	C19—H19A	0.9800
C4—H4A	0.9500	C19—H19B	0.9800
C5—C6	1.380 (4)	C19—H19C	0.9800
C5—H5A	0.9500	C20—H20A	0.9800
C6—C7	1.410 (3)	C20—H20B	0.9800
C6—H6A	0.9500	C20—H20C	0.9800
C7—C8	1.461 (3)	C21—C22	1.457 (5)
C8—C9	1.336 (3)	C22—C23	1.327 (3)
C8—H8A	0.9500	C22—H22A	0.9500
C9—C10	1.467 (3)	C23—C24	1.500 (3)
C9—H9A	0.9500	C23—H23A	0.9500
			
C1—O1—H1O	107.3 (17)	C14—C13—H13A	120.1
C21—O2—H2O	112 (3)	C13—C14—C15	121.80 (18)
C19—N1—C20	111.58 (15)	C13—C14—H14A	119.1
C19—N1—C18	112.43 (13)	C15—C14—H14A	119.1
C20—N1—C18	113.37 (14)	C14—C15—C10	118.40 (15)
C19—N1—H1N	107.7 (14)	C14—C15—C1	119.49 (14)
C20—N1—H1N	104.6 (15)	C10—C15—C1	122.08 (15)
C18—N1—H1N	106.5 (14)	C17—C16—C1	113.39 (13)
O1—C1—C2	106.28 (13)	C17—C16—H16A	108.9
O1—C1—C15	109.89 (13)	C1—C16—H16A	108.9
C2—C1—C15	108.87 (12)	C17—C16—H16B	108.9
O1—C1—C16	108.44 (12)	C1—C16—H16B	108.9
C2—C1—C16	113.44 (13)	H16A—C16—H16B	107.7
C15—C1—C16	109.84 (13)	C18—C17—C16	108.65 (13)
C3—C2—C7	119.14 (16)	C18—C17—H17A	110.0
C3—C2—C1	119.49 (16)	C16—C17—H17A	110.0
C7—C2—C1	121.36 (15)	C18—C17—H17B	110.0
C4—C3—C2	121.2 (2)	C16—C17—H17B	110.0
C4—C3—H3A	119.4	H17A—C17—H17B	108.3
C2—C3—H3A	119.4	N1—C18—C17	114.97 (13)
C5—C4—C3	120.2 (2)	N1—C18—H18A	108.5
C5—C4—H4A	119.9	C17—C18—H18A	108.5
C3—C4—H4A	119.9	N1—C18—H18B	108.5
C4—C5—C6	119.49 (19)	C17—C18—H18B	108.5
C4—C5—H5A	120.3	H18A—C18—H18B	107.5
C6—C5—H5A	120.3	N1—C19—H19A	109.5
C5—C6—C7	121.8 (2)	N1—C19—H19B	109.5
C5—C6—H6A	119.1	H19A—C19—H19B	109.5
C7—C6—H6A	119.1	N1—C19—H19C	109.5
C6—C7—C2	118.20 (19)	H19A—C19—H19C	109.5
C6—C7—C8	116.77 (18)	H19B—C19—H19C	109.5
C2—C7—C8	125.02 (16)	N1—C20—H20A	109.5
C9—C8—C7	127.72 (17)	N1—C20—H20B	109.5
C9—C8—H8A	116.1	H20A—C20—H20B	109.5
C7—C8—H8A	116.1	N1—C20—H20C	109.5
C8—C9—C10	126.48 (17)	H20A—C20—H20C	109.5
C8—C9—H9A	116.8	H20B—C20—H20C	109.5
C10—C9—H9A	116.8	O3—C21—O2	121.5 (3)
C11—C10—C15	118.57 (17)	O3—C21—C22	118.4 (4)
C11—C10—C9	117.12 (16)	O2—C21—C22	120.1 (2)
C15—C10—C9	124.18 (16)	C23—C22—C21	131.7 (3)
C12—C11—C10	121.86 (18)	C23—C22—H22A	114.2
C12—C11—H11A	119.1	C21—C22—H22A	114.2
C10—C11—H11A	119.1	C22—C23—C24	129.8 (3)
C11—C12—C13	119.46 (18)	C22—C23—H23A	115.1
C11—C12—H12A	120.3	C24—C23—H23A	115.1
C13—C12—H12A	120.3	O5—C24—O4	123.40 (18)
C12—C13—C14	119.77 (19)	O5—C24—C23	115.6 (2)
C12—C13—H13A	120.1	O4—C24—C23	120.98 (19)
			
O1—C1—C2—C3	−1.1 (2)	C12—C13—C14—C15	−1.6 (3)
C15—C1—C2—C3	−119.38 (16)	C13—C14—C15—C10	−1.3 (3)
C16—C1—C2—C3	118.00 (16)	C13—C14—C15—C1	176.88 (17)
O1—C1—C2—C7	−179.70 (14)	C11—C10—C15—C14	4.0 (2)
C15—C1—C2—C7	61.98 (19)	C9—C10—C15—C14	−171.64 (16)
C16—C1—C2—C7	−60.64 (19)	C11—C10—C15—C1	−174.11 (15)
C7—C2—C3—C4	−0.1 (3)	C9—C10—C15—C1	10.2 (2)
C1—C2—C3—C4	−178.74 (17)	O1—C1—C15—C14	−0.9 (2)
C2—C3—C4—C5	0.8 (3)	C2—C1—C15—C14	115.13 (16)
C3—C4—C5—C6	−0.2 (3)	C16—C1—C15—C14	−120.10 (16)
C4—C5—C6—C7	−1.0 (3)	O1—C1—C15—C10	177.21 (14)
C5—C6—C7—C2	1.7 (3)	C2—C1—C15—C10	−66.78 (19)
C5—C6—C7—C8	−177.35 (19)	C16—C1—C15—C10	57.99 (18)
C3—C2—C7—C6	−1.1 (2)	O1—C1—C16—C17	58.28 (17)
C1—C2—C7—C6	177.53 (15)	C2—C1—C16—C17	−59.54 (18)
C3—C2—C7—C8	177.82 (17)	C15—C1—C16—C17	178.38 (13)
C1—C2—C7—C8	−3.5 (3)	C1—C16—C17—C18	−164.67 (13)
C6—C7—C8—C9	145.99 (19)	C19—N1—C18—C17	61.9 (2)
C2—C7—C8—C9	−33.0 (3)	C20—N1—C18—C17	−65.79 (19)
C7—C8—C9—C10	−1.2 (3)	C16—C17—C18—N1	178.82 (13)
C8—C9—C10—C11	−144.80 (19)	O3—C21—C22—C23	−173.0 (3)
C8—C9—C10—C15	30.9 (3)	O2—C21—C22—C23	6.2 (5)
C15—C10—C11—C12	−4.1 (3)	C21—C22—C23—C24	−0.1 (5)
C9—C10—C11—C12	171.89 (17)	C22—C23—C24—O5	170.5 (3)
C10—C11—C12—C13	1.2 (3)	C22—C23—C24—O4	−8.0 (4)
C11—C12—C13—C14	1.7 (3)		

### Hydrogen-bond geometry (Å, °)

**Table:**

*D*—H···*A*	*D*—H	H···*A*	*D*···*A*	*D*—H···*A*
O1—H1O···O3^i^	0.83 (2)	1.95 (2)	2.770 (2)	173 (2)
O2—H2O···O4	0.89 (2)	1.56 (2)	2.442 (2)	171 (4)
N1—H1N···O5	0.88 (2)	1.80 (2)	2.6797 (19)	172 (2)
N1—H1N···O4	0.88 (2)	2.69 (2)	3.340 (2)	131.(2)
C16—H16B···O3^i^	0.99	2.63	3.267 (3)	122.
C19—H19A···O3^ii^	0.98	2.55	3.452 (3)	154.
C20—H20A···O3^ii^	0.98	2.94	3.781 (4)	144.
C9—H9A···O4^iii^	0.95	2.82	3.675 (2)	151.
C12—H12A···O4^iv^	0.95	2.62	3.460 (3)	148.
C17—H17A···O5^v^	0.99	2.92	3.865 (2)	159.
C20—H20B···O5^v^	0.98	2.39	3.296 (3)	154.

Symmetry codes: (i) −*x*, *y*−1/2, −*z*+1; (ii) *x*+1, *y*, *z*; (iii) *x*, *y*, *z*−1; (iv) −*x*, *y*−1/2, −*z*; (v) −*x*+1, *y*−1/2, −*z*+1.
